# Supplementary material for: Surface display for metabolic engineering of industrially important acetic acid bacteria
Source: PeerJ. 2018 Apr 6;6:e4626. doi: 10.7717/peerj.4626 (PMC5890722; doi:10.7717/peerj.4626)
Supplement: Supplemental Information 2 [file peerj-06-4626-s002.docx]

#required packages:

#install.packages("dplyr")

#install.packages("ggplot2")

#install.packages("growthcurver")

#install.packages("plyr")

#install.packages("multcomp")

#install.packages("reshape2")

##Fig 1

setwd("to file path")

Ecoli=read.csv('Ecoli.csv',header=TRUE)

library(reshape2)

EcoliMelt<-melt(Ecoli)

#1) To create and name a tiff file with desired width, height, and resolution (dpi):

tiff('Fig1_Ecoli.tiff', units="in", width=3, height=5, res=600)

#2) Creates the plot to be exported as a tiff:

boxplot(Ecoli, las=2, ylab=expression(bold(paste(Delta,'A'[405]*'/h×OD'[600]))),

par(mar=c(9.5,4,2.5,0.5),par(mgp=c(2.5,1,0))))

mtext(bold(bolditalic(E.~coli)~Strain)~"(N=8)",side=1,line=8)

groups<-c('a','c','a','b')

mtext(groups, at=c(1,2,3,4),side=3,line=0.75)

axis(side=3,label=NA,at=c(1,2,3,4,6,7,8,9,11,12,13,14))

#3) Exports the tiff file:

dev.off()

#To make a table summarizing data:

library(plyr)

ddply(EcoliMelt, c("variable"), summarise, Mean = mean(value), SD = sd(value),

SEM = sd(value)/sqrt(length(value)))

AnovaEcoli<-aov(value~variable,data=EcoliMelt)

TukeyHSD(AnovaEcoli,conf.level = 0.95)

library(multcomp)

tuk <- glht(AnovaEcoli, linfct = mcp(variable= "Tukey"))

summary(tuk)

cld(tuk)

#Fig 2

setwd("to file path")

GoxLinker<-read.csv("GoxLinker.csv",header=TRUE)

library(reshape2)

GoxLinkermelt<-melt(GoxLinker)

#1) To create and name a tiff file with desired width, height, and resolution (dpi):

tiff('Fig5_GoxLinker.tiff', units="in", width=4, height=5, res=600)

#2) Creates the plot to be exported as a tiff:

boxplot(GoxLinker, las=2, ylim=c(0,0.7), ylab=expression(bold(paste(Delta,'A'[405]*'/h×OD'[600]))),

par(mar=c(11,4,2.5,0.5), par(mgp=c(2.5,1,0))))

stripchart(value ~ variable, vertical =TRUE, data = GoxLinkermelt, method='overplot',

add = TRUE, pch = 20, col = 'black')

mtext(bold(bolditalic(G.~oxydans)~Strain)~"(N=12)",side=1,line=9.7)

groups<-c('b','b','c','b','a')

mtext(groups, at=c(1,2,3,4,5),side=3,line=0.75)

axis(side=3,label=NA,at=c(1,2,3,4,5))

#3) Exports the tiff file:

dev.off()

#Statistical analyses:

AnovaGoxLinker<-aov(value~variable,data=GoxLinkermelt)

summary(AnovaGoxLinker)

TukeyHSD(AnovaGoxLinker,conf.level = 0.95)

#Assign data to statistical groups:

library(multcomp)

tuk <- glht(AnovaGoxLinker, linfct = mcp(variable= "Tukey"))

summary(tuk)

cld(tuk)

#Fig 3

setwd("to file path")

Cleavable<-read.csv('Cleavable.csv',header=TRUE)

library(reshape2)

Cleavablemelt<-melt(Cleavable)

#1) To create and name a tiff file with desired width, height, and resolution (dpi):

tiff('Fig3_Cleavable.tiff', units="in", width=3.5, height=5, res=600)

#2) Creates the plot to be exported as a tiff:

boxplot(Cleavable, las=1, ylim=c(0,0.22),outline=TRUE, ylab=expression(bold(paste(Delta,'A'[405]*'/h×OD'[600]))),

par(mar=c(4,4.5,2.5,0.5),par(mgp=c(3,1,0))))

stripchart(value ~ variable, vertical =TRUE, outline=FALSE, data = Cleavablemelt,

method='overplot', add = TRUE, pch = 20, col = 'black', outline=FALSE)

mtext(bold(Sample)~"(N=12)",side=1,line=2.5)

mtext("p<0.001", at=c(1.5),side=3,line=-2)

#3) Exports the tiff file:

dev.off()

#To make a table summarizing data:

library(plyr)

ddply(Cleavablemelt, c("variable"), summarise, Mean = mean(value), SD = sd(value),

SEM = sd(value)/sqrt(length(value)), Median=median(value))

#Statistical analyses:

CleavableANOVA<-aov(value~variable,data=Cleavablemelt)

summary(CleavableANOVA)

TukeyHSD(CleavableANOVA,conf.level = 0.95)

x<-c(0.13528889, 0.12533333, 0.12871111, 0.14275556, 0.11265597, 0.11693405, 0.11283422, 0.10124777, 0.07898646, 0.08178244, 0.08055920, 0.08265618)

y<c(0.133333333,0.132977778,0.134755556,0.130311111,0.163814617,0.164705882,0.164705882,0.173262032,0.150808213, 0.153429445,0.155875928,0.156050677)

t.test(x,y)

#Assign data to statistical groups:

library(multcomp)

tuk <- glht(CleavableANOVA, linfct = mcp(variable= "Tukey"))

summary(tuk)

cld(tuk)

#Fig 4

setwd("to file path")

EcoliLinker<-read.csv("EcoliLinker.csv",header=TRUE)

library(reshape2)

EcoliLinkermelt<-melt(EcoliLinker)

#1) To create and name a tiff file with desired width, height, and resolution (dpi):

tiff('Fig4_EcoliLinker.tiff', units="in", width=5, height=5, res=600)

#2) Creates the plot to be exported as a tiff:

boxplot(EcoliLinker, las=2, ylim=c(0.00,1.40), ylab=expression(bold(paste(Delta,'A'[405]*'/h×OD'[600]))),

par(mar=c(11,4,3.5,0.5), par(mgp=c(2.5,1,0))))

stripchart(value ~ variable, vertical =TRUE, data = EcoliLinkermelt, method='overplot',

add = TRUE, pch = 20, col = 'black')

mtext(bold(bolditalic(E.~coli)~Strain)~"(N=12)",side=1,line=9.5)

groups<-c('b','b','a','a','c','b','d')

groups2<-c('c','d','c')

mtext(groups, at=c(1,2,3,4,5,6,7),side=3,line=0.75)

mtext(groups2, at=c(1,5,6),side=3,line=1.75)

axis(side=3,label=NA,at=c(1,2,3,4,5,6,7))

#3) Exports the tiff file:

dev.off()

#Statistical analyses:

AnovaEcoliLinker<-aov(value~variable,data=EcoliLinkermelt)

summary(AnovaEcoliLinker)

TukeyHSD(AnovaEcoliLinker,conf.level = 0.95)

#Assign data to statistical groups:

library(multcomp)

tuk <- glht(AnovaEcoliLinker, linfct = mcp(variable= "Tukey"))

summary(tuk)

cld(tuk)

#To make a table summarizing data:

library(plyr)

ddply(EcoliLinkermelt, c("variable"), summarise, Mean = mean(value), SD = sd(value),

SEM = sd(value)/sqrt(length(value)))

#Fig 5

setwd("to file path")

GoxLinker<-read.csv("GoxLinker.csv",header=TRUE)

library(reshape2)

GoxLinkermelt<-melt(GoxLinker)

#1) To create and name a tiff file with desired width, height, and resolution (dpi):

tiff('Fig5_GoxLinker.tiff', units="in", width=4, height=5, res=600)

#2) Creates the plot to be exported as a tiff:

boxplot(GoxLinker, las=2, ylim=c(0,0.7), ylab=expression(bold(paste(Delta,'A'[405]*'/h×OD'[600]))),

par(mar=c(11,4,2.5,0.5), par(mgp=c(2.5,1,0))))

stripchart(value ~ variable, vertical =TRUE, data = GoxLinkermelt, method='overplot',

add = TRUE, pch = 20, col = 'black')

mtext(bold(bolditalic(G.~oxydans)~Strain)~"(N=12)",side=1,line=9.7)

groups<-c('b','b','c','b','a')

mtext(groups, at=c(1,2,3,4,5),side=3,line=0.75)

axis(side=3,label=NA,at=c(1,2,3,4,5))

#3) Exports the tiff file:

dev.off()

#Statistical analyses:

AnovaGoxLinker<-aov(value~variable,data=GoxLinkermelt)

summary(AnovaGoxLinker)

TukeyHSD(AnovaGoxLinker,conf.level = 0.95)

#Assign data to statistical groups:

library(multcomp)

tuk <- glht(AnovaGoxLinker, linfct = mcp(variable= "Tukey"))

summary(tuk)

cld(tuk)

#Fig 6

setwd("to file path")

library(reshape2)

library(dplyr)

library(ggplot2)

# Read in the raw data and the platemap. You may need to first change your

# working directory with the setwd command.

data264 <- read.csv("264 GC.csv")

platemap264 <- read.csv("264 Platemap.csv")

# Reshape the data. Instead of rows containing the Time, Temperature,

# and readings for each Well, rows will contain the Time, Temperature, a

# Well ID, and the reading at that Well.

shape264 <- melt(data264, id=c("Time", "Temperature"), variable.name="Well", value.name="OD595")

# Add information about the experiment from the plate map. For each Well

# defined in both the reshaped data and the platemap, each resulting row

# will contain the absorbance measurement as well as the additional columns

# and values from the platemap.

annotated264 <- inner_join(shape264, platemap264, by="Well")

# Save the annotated data as a CSV for storing, sharing, etc.

write.csv(annotated264, "Gox Growth Curves 264 Annotated.csv")

conf_int95 <- function(data) {

n <- length(data)

error <- qt(0.975, df=n-1) * sd(data)/sqrt(n)

return(error)

}

# Group the data by the different experimental variables and calculate the

# sample size, average OD595, and 95% confidence limits around the mean

# among the replicates. Also remove all records where the Strain is NA.

stats264 <- annotated264 %>% group_by(Strain, Time) %>% summarise(N=length(OD595), Average=mean(OD595), CI95=conf_int95(OD595)) %>%filter(!is.na(Strain))

#1) To create and name a tiff file with desired width, height, and resolution (dpi):

tiff('Fig6_GrowthCurve.tiff', units="in", width=6, height=5, res=600)

#2) Creates the plot to be exported as a tiff:

ggplot(data=stats264, aes(x=Time, y=Average, color=Strain)) +

geom_ribbon(aes(ymin=Average-CI95, ymax=Average+CI95, fill=Strain),

color=NA, alpha=0.2)+ geom_line(show.legend = FALSE)+ theme_classic() + scale_x_continuous(breaks = c(0,100,200,300,400,500,600,700,800,900,1000,1100,1200,1300,1400)) +

labs(x=expression(bold("Time (min)")), y=expression(bold('Ln OD' [595])))+ scale_fill_discrete(breaks=c("Wildtype","p264-oprF-ST","p264-oprF-phoA","p264-oprF-FL1-phoA","p264-oprF-RL1-phoA", "p264-oprF-RL2-phoA","p264-oprF-RL3-phoA")) + theme(legend.position=c(0.2,0.7))

#3) Exports the tiff file:

dev.off()

#Fig 7

setwd("to file path")

DT=read.csv('DT.csv',header=TRUE)

library(reshape2)

DTmelt<-melt(DT)

#1) To create and name a tiff file with desired width, height, and resolution (dpi):

tiff('Fig7_DoublingTime.tiff', units="in", width=4, height=5, res=600)

#2) Creates the plot to be exported as a tiff:

boxplot(DT, las=2, ylab=expression(bold('Doubling time (min)')),

par(mar=c(10.5,4,3,0.5), mgp=c(2.5,1,0)))

stripchart(value ~ variable, vertical =TRUE, data = DTmelt,method='stack',

pch = 20, col = 'black', las=2, ylab=expression(bold('Doubling time (min)')),

ylim=c(0,150), add=FALSE)

mtext(bold(bolditalic(G.~oxydans)~Strain)~"(N=3)", side=1,line=9.5)

groups<-c('b','d','b','c','a','b','e')

mtext(groups, at=c(1,2,3,4,5,6,7),side=3,line=0.75)

axis(side=3,label=NA,at=c(1,2,3,4,5,6,7))

#3) Exports the tiff file:

dev.off()

#To make a table summarizing data:

library(plyr)

ddply(DTmelt, c("variable"), summarise, Mean = mean(value), SD = sd(value),

SEM = sd(value)/sqrt(length(value)))

#Statistical analyses:

AnovaDT<-aov(value~variable,data=DTmelt)

summary(AnovaDT)

TukeyHSD(AnovaDT,conf.level = 0.95)

#Assign data to statistical groups:

library(multcomp)

tuk <- glht(AnovaDT, linfct = mcp(variable= "Tukey"))

summary(tuk)

cld(tuk)
